# *Eucalyptus globulus* essential oil alleviates cold-induced ascites and physiological disturbances in broiler chickens

**DOI:** 10.1016/j.psj.2025.105720

**Published:** 2025-08-23

**Authors:** H. Mohebodini, V. Jazi, A. Ashayerizadeh, M.S. Salehi, A. Kahyani, E. Bidarnamani, F. Sharifi

**Affiliations:** a Department of Animal Sciences, University of Mohaghegh Ardabili, Ardabil 56199-11367, Iran; b Central Queensland Innovation and Research Precinct (CQIRP), Institute for Future Farming Systems, Central Queensland University, Rockhampton, QLD 4701, Australia; c Department of Animal Science, School of Agriculture, Shiraz University, Shiraz 71441-65186, Iran; d Department of Animal and Poultry Nutrition, Faculty of Animal Science, Gorgan University of Agricultural Sciences and Natural Resources, Gorgan 49189-4364, Iran; e Department of Physiology, School of Medicine, Isfahan University of Medical Sciences, Isfahan, Iran

**Keywords:** Antioxidant enzyme activity, Ascites, Broiler, Cold stress, *Eucalyptus globulus*

## Abstract

This study evaluated the efficacy of *Eucalyptus globulus* essential oil (EEO) supplementation in attenuating ascites incidence and physiological disturbances in broiler chickens exposed to a cold-induced pulmonary hypertension syndrome model. A total of 720 one-day-old male Ross 308 chicks were randomly assigned to six dietary treatments, each with six replicates of 20 birds. The thermoneutral control group received a basal diet under standard conditions (32°C on day 1, gradually reduced to 23°C by day 20 and maintained until day 42; relative humidity [RH] 50–60 %), whereas the remaining five groups were exposed to cold stress (temperature reduced by 4°C/day from day 11 until reaching 15°C, then maintained at 10–15°C until day 42, RH 55–65 %) to induce ascites, and were fed the basal diet supplemented with 0, 500, 1000, 1500, or 2000 mg/kg EEO. Cold stress decreased body weight gain and feed intake and increased ascites-related mortality (*P* < 0.05). It also elevated erythrocyte count, hemoglobin concentration, and hematocrit, while reducing pO₂ and O₂ saturation and inducing right ventricular hypertrophy (*P* < 0.05). In contrast, inclusion of EEO in the diet linearly improved body weight gain, partially restored hematological parameters, and favorably modulated blood gas values compared with the unsupplemented cold-stressed group (*P* < 0.05). Cold stress also suppressed superoxide dismutase (SOD) and glutathione peroxidase (GPx) activities in plasma and heart tissue, increased malondialdehyde (MDA) concentrations, and lowered plasma nitric oxide (NO) levels (*P* < 0.05). Dietary supplementation with EEO linearly increased SOD and GPx activities and plasma NO concentrations, while reducing MDA content (*P* < 0.05). Although EEO supplementation improved growth performance and physiological responses in cold-stressed broilers, values did not fully return to thermoneutral levels. Overall, these findings highlight the potential of EEO as a functional dietary additive to mitigate the adverse effects of cold-induced ascites and oxidative stress in broiler chickens.

## Introduction

Over the past few decades, substantial advances in broiler genetics and nutrition have markedly improved growth rates and feed efficiency (Korver, 2023). However, these gains have also increased the susceptibility of modern broiler strains to metabolic disorders, particularly pulmonary hypertension syndrome, commonly referred to as ascites (Miao et al., 2022). Ascites, characterized by the accumulation of serous fluid in the abdominal cavity, remains a leading cause of late-stage mortality and carcass condemnation in intensive poultry production systems, resulting in significant economic losses worldwide (Moreira et al., 2024).

The primary underlying cause of ascites in broilers is an imbalance between the elevated metabolic oxygen demands of fast-growing broilers and the limited capacity of their underdeveloped cardiopulmonary systems to meet these demands (Ahmadipour et al., 2025). Environmental stressors, particularly cold temperatures and hypobaric hypoxia at high altitudes, further exacerbate this imbalance by stimulating thermogenesis while simultaneously reducing oxygen availability (Khajali and Wideman, 2016). Such conditions trigger a series of compensatory physiological responses, including pulmonary vasoconstriction, increased erythropoiesis, elevated blood viscosity, and right ventricular hypertrophy, which may ultimately progress to right-sided heart failure, hepatic congestion, and ascitic fluid accumulation (Bi et al., 2024).

In addition to hemodynamic stress, oxidative stress has emerged as a key pathological factor in the development and progression of ascites (Arab et al., 2006; Miao et al., 2022). Hypoxia-induced oxidative stress results in excessive production of reactive oxygen species, which compromise vascular endothelial integrity, drive lipid peroxidation, and reduce nitric oxide (NO) bioavailability, thereby increasing pulmonary resistance and cardiac strain (Hassanpour et al., 2015; Khajali, 2022). Although synthetic antioxidants have been applied to counteract oxidative stress, concerns regarding their safety, residue accumulation, and consumer acceptance have heightened interest in natural alternatives (Khan et al., 2023). Among these, plant-derived compounds are of particular interest due to their multifunctional biological activities, including potent antioxidant potential.

*Eucalyptus globulus*, a member of the Myrtaceae family, is considered one of the most promising phytogenic candidates owing to its diverse pharmacological properties. Its principal bioactive constituent, 1,8-cineole (eucalyptol), possesses potent antioxidant, antimicrobial, anti-inflammatory, and vasorelaxant activities (Jun et al., 2013; Hoch et al., 2023). Traditionally, *Eucalyptus globulus* essential oil (EEO) has been used to manage respiratory disorders such as the common cold, bronchitis, sinusitis, and chronic obstructive pulmonary disease (Shiekh et al., 2024). Preclinical studies in hypertensive rat models have demonstrated that *Eucalyptus globulus* extract significantly lowers systemic blood pressure and induces vasorelaxation, primarily through NO-mediated pathways (Ajebli and Eddouks, 2021). This finding is further supported by Murad and Alqurashi (2024), who observed elevated NO levels and upregulated endothelial nitric oxide synthase (eNOS) expression, underscoring the potential of *Eucalyptus* as an antihypertensive agent to mitigate cardiovascular complications. Moreover, studies in broiler chickens have shown that EEO enhances the activities of key antioxidant enzymes, including superoxide dismutase (SOD), catalase, and glutathione peroxidase (GPx), while reducing oxidative biomarkers such as malondialdehyde (MDA) (Mohebodini et al., 2021).

Despite its promising pharmacological properties, the potential use of EEO as a dietary supplement to alleviate ascites in broiler chickens subjected to cold stress remains largely unexplored. Therefore, this study aimed to evaluate the efficacy of dietary EEO supplementation in alleviating cold-induced ascites in broiler chickens by assessing growth performance, right ventricular hypertrophy, antioxidant and biochemical responses, and ascites-related mortality.

## Materials and methods

All experimental procedures were conducted in accordance with the guidelines of the Institutional Animal Care and Use Committee of the University of Mohaghegh Ardabili (Ardabil, Iran), and ethical approval was obtained prior to the initiation of the study.

### Preparation and characterization of EEO

The essential oil used in this study was extracted and characterized as described in our previous work (Mohebodini et al., 2021). Fresh *Eucalyptus globulus* leaves were collected from Kivi City (Ardabil Province, Iran), air-dried at 25 ± 2°C for 72 h in a shaded, well-ventilated area, and subjected to hydrodistillation in a Clevenger-type apparatus for 3 h. The extracted oil was stored in dark glass bottles at 4°C until analysis. Chemical composition was determined by GC–MS (Agilent 7890 GC coupled with a 5975C MSD; Agilent Technologies, Santa Clara, CA) using an HP-5MS capillary column. The principal constituents were 1,8-cineole (67.85 %), α-pinene (12.69 %), α-terpineol (5.37 %), α-phellandrene (4.15 %), p-cymene (2.94 %), limonene (1.60 %), α-terpinene (1.48 %), and γ-terpinene (1.23 %) (Table 1).

**Table 1:** Chemical composition of *Eucalyptus globulus* essential oil.

No	Compound^1^	%^2^	RI (lit.)^3^	RI (calc.)^4^
1	α-Pinene	12.69	939	936
2	α-Phellandrene	4.15	1002	1006
3	α-Terpinene	1.48	1017	1019
4	p-Cymene	2.94	1026	1031
5	1,8-Cineole	67.85	1031	1035
6	Limonene	1.60	1032	1036
7	γ-Terpinene	1.23	1059	1062
8	α-Terpineol	5.37	1195	1197

1 Identified compounds.

2 percent amounts of identified compounds.

3 literature values of retention indices on the HP-5MS column.

4 calculated values of retention indices on the HP-5MS column.

### Birds, housing, and experimental design

A total of 720 one-day-old Ross 308 broiler chicks were obtained from a commercial hatchery. Upon arrival, all chicks were weighed and randomly allocated to six experimental treatments, each comprising six replicate pens with 20 birds per pen, in a completely randomized design. The birds were housed in an environmentally controlled poultry facility, with fresh wood shavings in each pen to maintain appropriate litter conditions. During the 42-day feeding trial, all birds had ad libitum access to fresh water and feed in mash form. The experimental design included one thermoneutral control group receiving a basal diet, and five cold-stressed groups receiving diets supplemented with EEO at 0, 500, 1000, 1500, or 2000 mg/kg. Based on the chemical composition of EEO (Table 1), in which 1,8-cineole represented 67.85 % of the oil, the dietary supplementation levels were equivalent to approximately 339, 678, 1018, and 1357 mg/kg of 1,8-cineole, respectively. Basal diets were formulated to meet or exceed the nutritional requirements specified in the Ross 308 Broiler Nutrition Specifications (Aviagen, 2019) for each growth phase: starter (days 0-10), grower (days 11-24), and finisher (days 25-42). The ingredient composition and calculated nutrient content of the basal diets are presented in Table 2. To prepare the dietary treatments, measured amounts of EEO were first blended with a portion of the basal diet to create a premix, which was subsequently mixed with the remaining basal diet to ensure homogeneity. The thermoneutral group was managed according to the Ross 308 Broiler Management Handbook, with ambient temperature set at 32°C on day 1, gradually reduced to 23°C by day 20, and maintained thereafter (relative humidity [RH]: 50-60 %). For the cold-stressed groups, ambient temperature was decreased from day 11 onward by 4°C per day until reaching 15°C, and then maintained between 10 and 15°C until day 42 (RH: 55-65 %) to induce ascites. Lighting was also applied according to the Ross 308 Broiler Management Handbook.

**Table 2:** Ingredient composition and calculated nutrient levels of the experimental diets.

Ingredients, g/kg	Starter phase (0-10 d)	Grower phase (11-24 d)	Finisher phase (25-42 d)
Corn	519.2	561.1	592.8
Soybean meal	385.0	363.5	326.8
Corn gluten meal	28.6	0.0	0.0
Sunflower oil	22.7	35.0	43.3
Limestone	8.0	7.1	6.5
Dicalcium phosphate	21.4	19.2	17.1
Sodium chloride	3.0	3.0	3.0
Sodium bicarbonate	0.8	0.9	1.0
Vitamin premix^1^	2.5	2.5	2.5
Mineral premix^2^	2.5	2.5	2.5
DL-methionine	3.1	3.0	2.7
L- lysine HCL	2.4	1.5	1.2
L-threonine	0.8	0.7	0.6
Total	1000.0	1000.0	1000.0
Calculated composition			
Metabolizable energy (MJ/kg)	12.34	12.77	13.18
Crude protein, %	23.04	20.56	19.14
Lysine, %	1.24	1.10	1.00
Methionine, %	0.62	0.57	0.52
Methionine + Cysteine, %	0.92	0.84	0.78
Threonine, %	0.81	0.73	0.67
Calcium, %	0.96	0.87	0.79
Available Phosphorus, %	0.48	0.44	0.40
Sodium, %	0.16	0.16	0.16
Chloride, %	0.23	0.23	0.23
Analyzed Composition			
Dry matter, %	91.58	91.49	91.53
Crude protein, %	22.57	20.52	18.55
Ether extract, %	6.02	7.01	7.51
Calcium, %	1.05	0.95	0.85
Total phosphorus, %	0.72	0.66	0.60

1 Vitamin premix provided the following per kilogram of diet: vitamin A, 11,500 IU; cholecalciferol, 2,100 IU; vitamin E, 22 IU; vitamin K3, 1.50 mg; thiamine, 3 mg; riboflavin, 4.4 mg; pantothenic acid, 25 mg; niacin, 40 mg; choline chloride, 560 mg; biotin, 0.1 mg; folic acid, 0.8 mg; pyridoxine 10 mg; vitamin B12, 0.060 mg.

2 Trace mineral premix provided the following in milligrams per kilogram of diet: iron, 50 mg; zinc, 55 mg; manganese, 75 mg; iodine, 1.8 mg; copper, 8 mg; selenium, 0.3 mg; cobalt, 0.2 mg.

### Measurements

Feed intake (FI) and body weight were recorded at the end of each growth phase. Body weight gain (BWG), FI, and feed conversion ratio (FCR) were calculated for the starter, grower, and finisher periods. Mortality was recorded daily, and FCR values were corrected for mortality. All deceased birds underwent necropsy to determine the cause of death. Ascites-related mortality was diagnosed based on characteristic pathological signs, including abdominal and pericardial fluid accumulation and an elevated right ventricle to total ventricular weight ratio (RV/TV) (Wideman et al., 2013). Birds with an RV/TV ratio ≥ 0.25 were classified as ascitic (Daneshyar et al., 2009).

At the end of the experiment, blood samples were collected from the brachial vein of two birds per pen and transferred into EDTA-coated tubes. Samples were immediately placed on ice and processed within one hour. Blood gas parameters and pH were measured using a blood gas analyzer (ABL 625, Radiometer, Copenhagen, Capital Region, Denmark). Hemoglobin concentration, hematocrit, and erythrocyte counts were determined using an automated hematology analyzer (Mindray BC-2800Vet, Mindray Bio-Medical Electronics Co., Ltd., Shenzhen, Guangdong, China). Moreover, a portion of blood was centrifuged at 1500× *g* for 10 minutes, and the resulting plasma was aliquoted and stored at −40°C until further analysis. The concentration of NO in plasma was determined as total nitrate and nitrite using the Griess reaction, following cadmium-mediated nitrate reduction as described by Behrooj et al. (2012). Plasma was deproteinized with ZnSO_4_ and NaOH, reduced with activated cadmium granules, and reacted with Griess reagents. Absorbance was measured at 540 nm using a UV-visible spectrophotometer (Cary 60 UV-Vis, Agilent Technologies, Santa Clara, CA).

Following blood collection, the same birds were euthanized by cervical dislocation in accordance with approved ethical protocols. The heart was promptly excised, rinsed with ice-cold phosphate-buffered saline, snap-frozen in liquid nitrogen, and stored at −80°C until further analysis. For antioxidant assays, right ventricular tissue samples were homogenized in ice-cold 0.05 M phosphate buffer (pH 7.4) containing 0.1 mM EDTA at a 1:10 (w/v) ratio using a glass-Teflon homogenizer. The homogenates were centrifuged at 1,500 × *g* for 15 minutes at 4°C, and the resulting supernatants were collected for enzymatic analysis. Previously collected plasma samples were also used to evaluate antioxidant status. SOD activity was determined by monitoring its ability to inhibit the reduction of reactive species involved in chemiluminescent reactions, with absorbance read at 560 nm, following the method described by Eriksson and Borg (1991). GPx activity was measured using a coupled enzyme system, where the oxidation of NADPH mediated by glutathione reductase in the presence of reduced glutathione and organic peroxide was measured at 340 nm, based on the protocol of Flohé and Günzler (1984). MDA content, indicative of lipid peroxidation, was assessed via a thiobarbituric acid reactive substances assay, detecting the formation of a colored adduct at 535 nm (Buege and Aust, 1978).

To count the bacterial population in the crop and cecum, approximately one g of digesta was aseptically collected from each bird. Samples were serially diluted in buffered peptone water, and 0.1 mL was plated onto de Man, Rogosa, and Sharpe agar for lactic acid bacteria (incubated anaerobically at 37.5°C for 24-48 h) and Eosin methylene blue agar for *Enterobacteriaceae* (incubated aerobically at 37.5°C for 24-48 h). Bacterial counts were expressed as log_10_ colony-forming units per gram of digesta (Ashayerizadeh et al., 2024). For pH measurement, 1 g of digesta was homogenized with 2 mL of distilled water, and the pH was determined using a digital pH meter (model K-21, NWKbinar, Germany).

### Statistical analysis

Data were first evaluated for normality using the Kolmogorov–Smirnov test. Subsequently, all variables were analyzed by one-way analysis of variance (ANOVA) within the General Linear Model (GLM) procedure of SAS software (Version 9.1; SAS Institute, Cary, NC, 2003). The experimental unit for all analyses was the pen. Treatment means were separated using Tukey’s post hoc test. In addition, linear and quadratic effects of graded EEO inclusion levels were examined using orthogonal polynomial contrasts. Statistical significance was declared at *P* < 0.05.

## Results

The effects of treatments on broiler performance are summarized in Table 3. During the starter phase (pre-stress period), no significant differences were observed among treatments for FI, BWG, or FCR. In contrast, exposure to cold stress during the grower phase significantly reduced FI and BWG compared with birds reared under thermoneutral conditions (*P* < 0.05), although FCR was unaffected. These detrimental effects persisted through the finisher phase and across the entire rearing period (*P* < 0.05). Dietary EEO supplementation exerted a linear, dose-dependent ameliorative effect, partially mitigating the negative impact of cold stress on growth performance (*P* < 0.05). Birds receiving higher EEO inclusion levels showed linearly improved FI and BWG compared with unsupplemented cold-stressed controls (*P* < 0.05); however, their performance remained significantly lower than that of birds maintained under thermoneutral conditions.

**Table 3:** Influence of eucalyptus essential oil (EEO) supplementation on growth performance of cold-stressed broiler chickens^1^.

	Experimental groups^2^							*P*-value		
Items	NT	CT+EEO_0_	CT+EEO_500_	CT+EEO_1000_	CT+EEO_1500_	CT+EEO_2000_	SEM	Model	Linear	Quadratic
Starter (0-10 d)										
BWG^3^, g	242	238	231	234	243	240	3.21	0.643	0.521	0.563
FI^4^, g	268	270	260	265	272	267	4.54	0.701	0.595	0.640
FCR^5^, g/g	1.11	1.13	1.12	1.14	1.12	1.11	0.03	0.685	0.603	0.697
Grower (11-24 d)										
BWG, g	857^a^	763^c^	785^bc^	790^bc^	816^ab^	827^ab^	13.65	<0.001	0.001	0.732
FI, g	1376^a^	1196^b^	1248^ab^	1243^ab^	1308^ab^	1341^a^	18.97	0.001	0.012	0.894
FCR, g/g	1.61	1.57	1.59	1.57	1.60	1.62	0.02	0.881	0.435	0.673
Finisher (25-42 d)										
BWG, g	1482^a^	1106^c^	1181^bc^	1250^bc^	1269^bc^	1295^b^	35.23	<0.001	0.005	0.891
FI, g	2994^a^	2489^d^	2622^cd^	2788^bc^	2780^bc^	2849^b^	24.98	<0.001	0.001	0.606
FCR, g/g	2.02	2.25	2.22	2.23	2.19	2.20	0.03	0.097	0.545	0.720
Overall (0-42 d)										
BWG, g	2581^a^	2107^d^	2197^cd^	2274^bc^	2328^bc^	2362^b^	28.40	<0.001	<0.001	0.853
FI, g	4638^a^	3955^d^	4130^cd^	4296^bc^	4360^bc^	4457^b^	21.67	<0.001	<0.001	0.781
FCR, g/g	1.80	1.88	1.88	1.90	1.87	1.89	0.01	0.875	0.701	0.895

1 Growth performance data represent the means of six replicate pens, each containing 20 broiler chickens.

2 NT: broilers in thermoneutral conditions fed a basal diet; CT+EEO_0_: broilers under cold stress fed a basal diet without eucalyptus essential oil; CT+EEO_1000_, CT+EEO_1500_, CT+EEO_2000_: broilers under cold stress fed basal diets supplemented with 500, 1000, 1500, or 2000 mg/kg eucalyptus essential oil, respectively.

3 Body weight gain.

4 Feed intake.

5 Feed conversion ratio.

a-d Means in the same row with different superscripts differ (*P* < 0.05).

As shown in Table 4, cold-stressed birds without EEO supplementation had the highest total and ascites-related mortality (*P* < 0.05). Mortality declined linearly with increasing dietary EEO inclusion (*P* < 0.05). The lowest mortality rate was recorded in the thermoneutral control group (*P* < 0.05).

**Table 4:** Influence of eucalyptus essential oil (EEO) supplementation on mortality and ascites-related indices in cold-stressed broiler chickens.

	Experimental groups^1^							*P*-value		
Items	NT	CT+EEO_0_	CT+EEO_500_	CT+EEO_1000_	CT+EEO_1500_	CT+EEO_2000_	SEM	Model	Linear	Quadratic
Ascites mortality, %	0.63^c^	18.75^a^	12.50^b^	11.20^b^	10.00^b^	8.13^b^	1.47	<0.001	<0.001	0.450
Non-ascites mortality, %	0.63	3.13	1.25	1.88	1.25	1.88	0.39	0.677	0.563	0.712
Total mortality, %	1.25^c^	21.88^a^	13.75^b^	13.08^b^	11.25^b^	10.01^b^	1.52	<0.001	<0.001	0.897
RV/TV ratio^2^	0.19^c^	0.31^a^	0.25^b^	0.26^b^	0.24^b^	0.24^b^	0.01	<0.001	<0.001	0.558

1 NT: broilers in thermoneutral conditions fed a basal diet; CT+ EEO_0_: broilers under cold stress fed a basal diet without eucalyptus essential oil; CT+EEO_1000_, CT+EEO_1500_, CT+EEO_2000_: broilers under cold stress fed basal diets supplemented with 500, 1000, 1500, or 2000 mg/kg eucalyptus essential oil, respectively.

2 Right ventricles to total ventricles ratio.

a-c Means in the same row with different superscripts differ (*P* < 0.05).

The data on blood gas parameters are reported in Table 5. Cold stress significantly increased the partial pressure of carbon dioxide (pCO₂) while decreasing the partial pressure of oxygen (pO₂) and oxygen saturation (O₂ saturation) in broiler chickens (*P* < 0.05). Dietary EEO supplementation linearly improved pO₂, O₂ saturation, and pCO₂ in cold-stressed birds (*P* < 0.05). Blood pH and bicarbonate concentrations were not affected by treatment.

**Table 5:** Influence of eucalyptus essential oil (EEO) supplementation on venous blood gas parameters and pH values in cold-stressed broiler chickens.

	Experimental groups^2^							*P*-value		
Items	NT	CT+EEO_0_	CT+EEO_500_	CT+EEO_1000_	CT+EEO_1500_	CT+EEO_2000_	SEM	Model	Linear	Quadratic
Partial pressure of O_2_, mmHg	61.2^a^	57.5^c^	57.9^c^	59.8^b^	60.7^ab^	61.6^a^	0.85	<0.001	<0.001	0.534
Partial pressure of CO_2_, mmHg	40.1^d^	49.8^a^	46.4^b^	45.1^b^	43.4^c^	41.0^d^	0.96	<0.001	<0.001	0.727
HCO_3_^−^, mmol/L	29.6	31.2	31.1	30.5	29.1	30.2	0.43	0.683	0.601	0.892
O_2_ saturation, %	68.9^a^	60.2^c^	62.6^b^	63.3^b^	65.9^ab^	68.3^a^	0.57	<0.001	0.001	0.548
pH	7.29	7.30	7.31	7.29	7.32	7.31	0.06	0.709	0.651	0.743

^1^NT: broilers in thermoneutral conditions fed a basal diet; CT+ EEO_0_: broilers under cold stress fed a basal diet without eucalyptus essential oil; CT+EEO_1000_, CT+EEO_1500_, CT+EEO_2000_: broilers under cold stress fed basal diets supplemented with 500, 1000, 1500, or 2000 mg/kg eucalyptus essential oil, respectively.

a-c Means in the same row with different superscripts differ (*P* < 0.05).

Table 6 presents that cold-stressed birds had significantly higher erythrocyte counts, hematocrit, and hemoglobin compared with thermoneutral controls (*P* < 0.05). These hematological alterations were linearly alleviated with increasing dietary EEO supplementation (*P* < 0.05). Cold stress also reduced plasma NO concentrations (*P* < 0.05), whereas dietary EEO linearly increased NO levels, though full restoration to thermoneutral values was not achieved.

**Table 6:** Influence of eucalyptus essential oil (EEO) supplementation on hematological parameters and nitric oxide concentration in cold-stressed broiler chickens.

	Experimental groups^1^							*P*-value		
Items	NT	CT+EEO_0_	CT+EEO_500_	CT+EEO_1000_	CT+EEO_1500_	CT+EEO_2000_	SEM	Model	Linear	Quadratic
Erythrocytes count, 10^6^/µL	2.50^c^	3.32^a^	3.19^a^	2.81^b^	2.54^c^	2.47^c^	0.08	<0.001	0.001	0.622
Hemoglobin, g/dL	8.92^c^	11.65^a^	11.05^ab^	10.57^b^	9.73^bc^	9.30^c^	0.23	<0.001	0.002	0.781
Hematocrit, % Nitric oxide, µmol/L	31.40^d^ 22.12^a^	42.60^a^ 9.78^d^	41.50^a^ 12.48^c^	38.71^b^ 12.29^c^	38.15^b^ 17.42^b^	35.00^c^ 18.02^b^	1.24 0.58	<0.001 <0.001	<0.001 <0.001	0.453 0.527
Erythrocytes count, 10^6^/µL	2.50^c^	3.32^a^	3.19^a^	2.81^b^	2.54^c^	2.47^c^	0.08	0.004	0.001	0.622

1 NT: broilers in thermoneutral conditions fed a basal diet; CT+ EEO_0_: broilers under cold stress fed a basal diet without eucalyptus essential oil; CT+EEO_1000_, CT+EEO_1500_, CT+EEO_2000_: broilers under cold stress fed basal diets supplemented with 500, 1000, 1500, or 2000 mg/kg eucalyptus essential oil, respectively.

a-c Means in the same row with different superscripts differ (*P* < 0.05).

Table 7 shows the antioxidant profiles of plasma and heart tissue in broilers under different treatments. Cold stress significantly reduced SOD and GPx activities while elevating MDA concentrations in both plasma and heart tissue (*P* < 0.05). Incremental dietary EEO supplementation linearly enhanced SOD and GPx activities and decreased MDA concentrations in both tissues (*P* < 0.05).

**Table 7:** Influence of eucalyptus essential oil (EEO) supplementation on antioxidant enzyme activities in the plasma and heart tissue of cold-stressed broiler chickens.

	Experimental groups^1^							*P*-value		
Items	NT	CT+EEO_0_	CT+EEO_500_	CT+EEO_1000_	CT+EEO_1500_	CT+EEO_2000_	SEM	Model	Linear	Quadratic
Plasma										
SOD, U/L	1138^a^	912^c^	1020^c^	1072^b^	1095^b^	1109^ab^	17.90	<0.001	<0.001	0.428
GPx, U/L	53.6^a^	38.5^d^	44.6^c^	46.5^bc^	48.0^b^	48.9^b^	1.20	<0.001	<0.001	0.611
MDA, nmol/L	0.51^d^	1.42^a^	1.10^b^	0.95^bc^	0.80^c^	0.66^cd^	0.04	<0.001	<0.001	0.002
Heart										
SOD, U/mg protein	87.5^a^	63.4^d^	69.6^c^	78.5^b^	79.1^b^	80.8^b^	1.86	<0.001	<0.001	0.568
GSH-Px, U/mg protein	36.4^a^	24.2^d^	27.5^c^	28.1^c^	32.2^b^	32.4^b^	0.58	<0.001	<0.001	0.771
MDA, nmol/mg protein	1.17^d^	2.10^a^	1.70^b^	1.65^b^	1.45^bc^	1.22^c^	0.03	<0.001	<0.001	0.003

1 NT: broilers in thermoneutral conditions fed a basal diet; CT+ EEO_0_: broilers under cold stress fed a basal diet without eucalyptus essential oil; CT+EEO_1000_, CT+EEO_1500_, CT+EEO_2000_: broilers under cold stress fed basal diets supplemented with 500, 1000, 1500, or 2000 mg/kg eucalyptus essential oil, respectively. ^2^SOD: superoxide dismutase; GSH-Px: glutathione peroxidase; MDA: malondialdehyde.

a-d Means in the same row with different superscripts differ (*P* < 0.05).

Table 8 summarizes the effects of treatments on pH and microbial population in the crop and cecum of broiler chickens. Cold stress significantly increased *Enterobacteriaceae* count while reducing lactic acid bacteria (LAB) population in both sites compared with the thermoneutral control group (*P* < 0.05). Incremental dietary EEO supplementation linearly decreased *Enterobacteriaceae* count and increased LAB populations in both the crop and cecum (*P* < 0.05). Crop and cecal pH values were not significantly influenced by treatments.

**Table 8:** Influence of eucalyptus essential oil (EEO) supplementation on pH and gut bacterial population of cold-stressed broiler chickens.

	Experimental groups^1^							*P*-value		
Items^2^	NT	CT+EEO_0_	CT+EEO_500_	CT+EEO_1000_	CT+EEO_1500_	CT+EEO_2000_	SEM	Model	Linear	Quadratic
Crop										
pH	4.61	4.58	4.59	4.57	4.56	4.58	0.10	0.753	0.594	0.681
LAB, log_10_ cfu/g	7.85^a^	6.30^e^	6.70^d^	7.05^c^	7.15^bc^	7.75^a^	0.08	<0.001	0.001	0.452
ENT, log_10_ cfu/g	5.20^d^	6.65^a^	6.30^ab^	5.95^c^	5.70^cd^	5.30^d^	0.06	<0.001	<0.001	0.601
Ceca										
pH	6.36	6.37	6.35	6.38	6.34	6.37	0.11	0.892	0.733	0.845
LAB, log_10_ cfu/g	7.95^a^	6.45^d^	6.60^cd^	7.25^b^	7.40^b^	8.90^a^	0.06	<0.001	<0.001	0.605
ENT, log_10_ cfu/g	7.01^d^	8.80^a^	8.59^ab^	8.15^c^	7.93^c^	7.08^d^	0.07	<0.001	0.001	0.673

1 NT: broilers in thermoneutral conditions fed a basal diet; CT+ EEO_0_: broilers under cold stress fed a basal diet without eucalyptus essential oil; CT+EEO_1000_, CT+EEO_1500_, CT+EEO_2000_: broilers under cold stress fed basal diets supplemented with 500, 1000, 1500, or 2000 mg/kg eucalyptus essential oil, respectively.

2 LAB: lactic acid bacteria; ENT: *Enterobacteriaceae*.

a-d Means in the same row with different superscripts differ (*P* < 0.05).

## Discussion

Ascites syndrome in broilers is a hypoxia-driven metabolic disorder arising from an imbalance between elevated oxygen demand and limited pulmonary capacity, particularly under cold environmental conditions. This imbalance leads to pulmonary hypertension, right ventricular hypertrophy, and abdominal fluid accumulation, ultimately impairing growth and increasing mortality (Wideman et al., 2013). The present study evaluated the efficacy of dietary supplementation with EEO in mitigating cold stress-induced ascites and its associated impacts on broiler performance. As expected, cold exposure during the grower and finisher phases significantly lowered FI and BWG, consistent with previous studies on the adverse impacts of cold stress on broiler growth performance and metabolism (Abolfathi et al., 2021; Zhou et al., 2021; Gao et al., 2025). This decline in performance is likely attributable to cold-induced anorexia and a metabolic shift that prioritizes thermogenesis over growth (Zamani Moghaddam et al., 2009; Kodambashi Emami et al., 2017). Cold stress also impairs intestinal function by disrupting mucosal integrity, altering tight junction protein expression, and inducing oxidative stress, thereby elevating intestinal permeability and compromising barrier function (Zhou et al., 2021; Liu et al., 2022). Hu and Guo (2008) and Rahmani et al. (2018) reported that cold stress–induced corticosterone elevation reduces villus height and increases crypt depth, changes that impair nutrient absorption and ultimately compromise feed efficiency and growth. In the current study, cold stress also increased ascites-related mortality and RV/TV ratio, indicating the induction of pulmonary hypertension. Dietary inclusion of EEO elicited dose-dependent improvements in broiler chickens' growth performance and ascites parameters, with birds receiving higher EEO levels showing significantly greater BWG and reduced mortality rates and RV/TV ratios compared with unsupplemented cold-stressed birds. Di et al. (2022) reported that dietary supplementation with 1,8-cineole, the principal constituent of EEO, increased average daily gain in broilers from days 22 to 42 and across the entire rearing period. The improved performance and ascites outcomes observed in this study may be linked to bioactive phytochemicals in EEO, including phenolics, terpenes, and flavonoids, which exert antioxidant, anti-inflammatory, antimicrobial, and antihypertensive effects (Ajebli and Eddouks, 2021; Hoch et al., 2023). Evidence also suggests that EEO enhances productivity by stimulating digestive enzyme activity, strengthening antioxidant defenses, stabilizing intestinal microbiota, preserving mucosal integrity, and reducing lipid peroxidation (Mashayekhi et al., 2018; Jiang et al., 2021; Mohebodini et al., 2021; Di et al., 2022). Comparable benefits have been reported for other essential oils, such as cinnamon and thyme, which improved performance in cold-stressed laying hens (Torki et al., 2015).

Efficient gas exchange and hematological stability are crucial for oxygen delivery and metabolic regulation in animals. In the present study, cold-stressed broilers exhibited increased pCO_2_ alongside reduced pO_2_ and O_2_ saturation, indicative of impaired alveolar ventilation and compromised gas exchange efficiency, ultimately leading to functional tissue hypoxemia (Hossain and Akter, 2022; Miao et al., 2022). These alternations triggered compensatory erythropoietic responses, evidenced by significant increases in erythrocyte counts, hematocrit, and hemoglobin concentration in cold-exposed birds. This adaptive mechanism is primarily mediated via the hypoxia-inducible factor-1 alpha (HIF-1α)/ erythropoietin signalling axis, which promotes erythropoiesis to restore oxygen-carrying capacity (Yoon et al., 2006; Wideman et al., 2013). While this adaptation is beneficial in the short term, its chronic persistence may result in polycythemia, elevated blood viscosity, and impaired microvascular perfusion, thereby exacerbating susceptibility to ascites and cardiopulmonary dysfunction (Hossain and Akter, 2022). Similarly, previous studies have shown that cold stress increases erythrocyte counts, hematocrit, hemoglobin concentration, and arterial pO_2,_ while reducing pO_2_ and O_2_ saturation (Rahmani et al., 2017; Fathi et al., 2022, 2023). The present findings showed that dietary supplementation with EEO significantly ameliorated these pathological changes in a dose-dependent manner. Birds receiving EEO showed linear improvements in pO_2_, O_2_ saturation, and pCO_2_ values, accompanied by reductions in erythrocyte count, hematocrit, and hemoglobin levels, reflecting systemic restoration of oxygen homeostasis. EEO, with 1,8-cineole as its major constituent, may mitigate cold stress-induced pathophysiology through antioxidant and vasoprotective actions (Ajebli and Eddouks, 2021). In broilers, dietary 1,8-cineole supplementation has been shown to enhance antioxidant enzyme activity and reduce lipid peroxidation, thereby preserving cellular and erythrocyte membrane integrity (Jiang et al., 2021; Di et al., 2022). Findings from mammalian models further demonstrate that 1,8-cineole increases endothelial NO bioavailability, which in turn supports red blood cell deformability, improves microvascular perfusion, and enhances cardiopulmonary resilience under stress conditions (Murad and Alqurashi, 2024).

The findings of this study demonstrate that cold stress significantly attenuates plasma NO levels in broiler chickens, indicating vascular dysfunction and endothelial impairment. NO, predominantly synthesized by eNOS, is essential for regulating pulmonary arterial tone via flow-dependent vasodilation (Hassanpour et al., 2015; Gonzalez et al., 2025). Under cold-induced hypoxic conditions, decreased expression and activity of eNOS, as reported by Wideman et al. (2013), impair this vasodilatory response, resulting in increased pulmonary vascular resistance and elevated susceptibility to pulmonary arterial hypertension and ascites. Concurrently, inducible NOS (iNOS), typically upregulated during inflammatory responses to sustain NO production, is downregulated following prolonged cold exposure and systemic hypoxia (Hassanzadeh et al., 2014; Miao et al., 2022). This dual suppression limits both the rapid and sustained NO synthesis, thereby compromising the pulmonary vasculature’s capacity to adapt to hemodynamic stress (Baghbanzadeh and Decuypere, 2008; Hassanpour et al., 2015). Moreover, cold-induced oxidative stress exacerbates endothelial dysfunction; increased reactive oxygen species (ROS) interact with NO to form peroxynitrite, a potent cytotoxic oxidant that depletes NO bioavailability and intensifies endothelial injury (Bautista-Ortega et al., 2014). The consequent reduction in NO further impairs vasodilation and promotes right ventricular overload, a key feature in the pathogenesis of ascites. Importantly, the present study showed that dietary supplementation with increasing doses of EEO linearly elevated plasma NO levels in cold-stressed broilers, indicating partial recovery of endothelial function. In support of these findings, Murad and Alqurashi (2024) reported that 1,8-cineole, the main monoterpene oxide in *Eucalyptus globulus*, increased plasma NO levels and upregulated aortic eNOS expression in a vasopressin-induced angina rat model. Similarly, Ajebli and Eddouks (2021) found that *Eucalyptus globulus* extract lowered blood pressure and induced vasorelaxation in Nω-Nitro-l-arginine methyl ester (L-NAME)-treated rats via NO-dependent pathways, independent of prostaglandin or calcium/adrenergic signalling. These data suggest a shared mechanism involving eNOS activation and attenuation of oxidative stress, highlighting EEO’s therapeutic potential for supporting vascular health under environmental stress.

Oxidative stress plays a pivotal role in the pathogenesis of cold-induced ascites in broiler chickens by disrupting redox haemostasis through excessive production of ROS, which initiates lipid peroxidation, damages cellular structures, and impairs physiological function (Arab et al., 2006; Mohebodini et al., 2019). In the present study, cold stress significantly compromised the antioxidant capacity in the plasma and heart samples, as indicated by decreased activities of SOD and GPx, along with increased levels of MDA. These findings align with previous reports highlighting cardiac and hepatic tissue vulnerability to cold-induced oxidative stress (Abolfathi et al., 2021; Fathi et al., 2023). Moreover, the increase in MDA content, a marker of lipid peroxidation, was positively associated with the RV/TV ratio, supporting its involvement in the pathogenesis of pulmonary hypertension syndrome (Pan et al., 2005; Kodambashi Emami et al., 2017). Such oxidative perturbations may impair myocardial function and contribute to right ventricular hypertrophy (Wei et al., 2024). The present findings indicate that dietary EEO inclusion dose-dependently enhanced SOD and GPx activities while reducing MDA concentrations in plasma and heart, indicating partial restoration of systemic redox homeostasis. Consistent with this, our previous work under non-challenged conditions showed that EEO supplementation enhanced antioxidant enzyme activities and reduced lipid peroxidation in both serum and liver of broilers (Mohebodini et al., 2021). In agreement, Di et al. (2022) reported that dietary 1,8-cineole increased SOD and GPx activities while decreasing MDA concentrations in broiler serum and liver. These protective effects are attributed to the bioactive constituents of EEO, which possess ROS-scavenging capacity and activate redox-sensitive signaling pathways, most notably the nuclear factor erythroid 2-related factor 2 (Nrf2) axis (Porres-Martínez et al., 2016; Di et al., 2022).

In the present study, cold-induced stress significantly disrupted gastrointestinal microbial balance in broiler chickens, as indicated by increased *Enterobacteriaceae* count and reduced population of LAB in the crop and ceca. Consistent with these observations, previous studies demonstrated that cold exposure enhances cecal colonization by *Escherichia coli* while reducing populations of beneficial *Lactobacillus* spp., highlighting the vulnerability of the gut microbiota to low ambient temperatures (Rahmani et al., 2018; Liu et al., 2022). Cold stress induces dysbiosis in poultry through neuroendocrine and oxidative pathways (Tsiouris et al., 2015; Gao et al., 2025). Elevated corticosterone and altered catecholamine-serotonin signaling disrupt microbial balance, while excessive reactive oxygen species compromise redox homeostasis and mucosal immunity (Rahmani et al., 2018; Lyte et al., 2024). These disturbances impair tight junction integrity and reduce short-chain fatty acid production, resulting in an increased proliferation of opportunistic bacteria (Gao et al., 2025). In this study, dietary supplementation with EEO effectively counteracted the adverse effects of cold stress, as evidenced by a linear reduction in *Enterobacteriaceae* counts and an increase in LAB populations. In vitro studies have demonstrated that EEO possesses strong antimicrobial activity against pathogenic *Escherichia coli* and *Salmonella typhimurium* (Muhizi et al., 2025). Jiang et al. (2021) reported that dietary supplementation with 1,8-cineole increased cecal *Lactobacillus* population, reduced *Salmonella* count, and alleviated heat stress-induced inflammation in broilers by restoring microbial homeostasis. Previous studies have also reported effects consistent with our findings, showing that phytogenic compounds such as curcumin reduced the population of *Escherichia coli* while increasing *Lactobacillus* count in the ceca of cold-stressed broiler chickens (Rahmani et al., 2018). The antimicrobial activity of EEO is mainly attributed to its bioactive compounds, 1,8-cineole, α-pinene, and limonene, which disrupt bacterial membranes and increase permeability (Merghni et al., 2023). Phenolic constituents may also further promote beneficial bacteria through prebiotic-like effects and enhance mucosal defense via mucin secretion (Zeng et al., 2015; Shirani et al., 2019). These actions suggest symptomatic alleviation of dysbiosis by suppressing pathogens and enriching commensals. However, further studies are required to clarify whether EEO also influences host stress-immune regulatory pathways underlying cold stress-induced dysbiosis.

In summary, dietary supplementation with EEO represents a promising phytogenic strategy to improve growth performance, enhance physiological resilience, and mitigate ascites incidence in broilers exposed to cold stress. These protective effects are largely attributed to the antioxidant, antihypertensive, and antimicrobial properties of its bioactive constituents, particularly 1,8-cineole. Overall, the findings highlight the potential of EEO as a multifunctional feed additive to support poultry health and productivity under environmentally challenging conditions.

## CRediT authorship contribution statement

**H. Mohebodini:** Conceptualization, Data curation, Formal analysis, Funding acquisition, Investigation, Methodology, Project administration, Resources, Software, Supervision. **V. Jazi:** Writing – original draft, Writing – review & editing. **A. Ashayerizadeh:** Data curation, Formal analysis, Funding acquisition, Investigation, Project administration, Resources, Software, Supervision, Validation, Visualization. **M.S. Salehi:** Data curation, Formal analysis, Methodology, Software. **A. Kahyani:** Investigation, Methodology, Project administration, Validation, Visualization. **E. Bidarnamani:** Conceptualization, Data curation, Formal analysis, Software, Validation, Visualization. **F. Sharifi:** Writing – original draft, Writing – review & editing.

## Disclosures

The authors declare that there is no conflict of interest.
